# Demonstrating Tactical Combat Casualty Care in Simulated Environments to Enable Passive, Autonomous Documentation: Protocol for a Prospective Simulation-Based Study

**DOI:** 10.2196/67673

**Published:** 2025-03-17

**Authors:** Jeanette R Little, Triana Rivera-Nichols, Holly H Pavliscsak, Omar Badawi, James C Gaudaen, Chevas R Yeoman, Todd S Hall, Ethan T Quist, Ericka L Stoor-Burning

**Affiliations:** 1 The Telemedicine and Advanced Technology Research Center Fort Detrick, MD United States

**Keywords:** tactical combat casualty care, TCCC, automation, medical documentation, DD form 1380, combat casualty care, artificial intelligence, AI, machine learning, ML, point of injury, POI, simulation, military health, passive data collection, sensors, algorithms, medical record

## Abstract

**Background:**

The Telemedicine & Advanced Technology Research Center (TATRC) commenced a new research portfolio specifically addressing Autonomous Casualty Care (AC2) in 2023. The first project within this portfolio addresses the current and historical challenges of capturing tactical combat casualty care (TCCC) data in operational settings.

**Objective:**

The initial autonomous casualty care effort, the Passive Data Collection using Autonomous Documentation research project, conducts systematic, simulated patient and casualty care scenarios, leveraging suites of passive sensor inputs to populate a data repository that will automate future combat care.

**Methods:**

To obtain the required datasets, TATRC will engage care provider participants who provided consent in one of 6 randomized simulated TCCC scenarios leveraging an institutional review board–approved office protocol (#M-11057). These simulations will leverage mannikins (low and high fidelity) and live simulated patients (eg, human actors who provided consent). All consenting participants (eg, both the care providers and live simulated patients) will be equipped with suites of sensors that will passively collect data on care delivery actions and patient physiology. Simulated data is being collected at Fort Detrick, Maryland; Fort Sam Houston, Texas; Fort Indiantown Gap, Pennsylvania; Fort Liberty, North Carolina; and a commercial site in Greenville, North Carolina.

**Results:**

Across all research locations, TATRC will collect and annotate approximately 2500 simulation procedures tasks by March 2025. These study data will generate the first machine learning and artificial intelligence algorithms to populate Department of Defense (DD) Form 1380 fields accurately and reliably. Additional data collected past March 2025 will be used to continue to refine and mature the algorithm.

**Conclusions:**

The military health care system (MHS) lacks real-world datasets for TCCC care at the point of injury. Developing a data repository of simulated TCCC data is required as an essential step toward automating TCCC care. If TATRC’s research efforts result in the ability to automate care delivery documentation, this will alleviate the cognitive burden of TCCC care providers in austere, chaotic environments. By generating a TCCC data repository through this Autonomous Documentation research project, TATRC will have opportunities to leverage this research data to create machine learning and artificial intelligence models to advance passive, automated medical documentation across the health care continuum.

**International Registered Report Identifier (IRRID):**

DERR1-10.2196/67673

## Introduction

The military health care system (MHS) is a Joint Service health care system that operates under the Defense Health Agency (DHA) [1]. Within this health care system, there is a diversified set of health care data ranging from routine preventative services for service members (SMs) and their families to battlefield casualty statuses. Due to the nature of its complexity, the MHS thrives in stable connected environments and languishes in operational settings where network communications are compromised or insecure. Thus, when our SMs are located in various tactical environments in multidomain operations, the MHS lacks a robust, accurate, and reliable methodology to collect, store, and track health data, often referred to as tactical combat casualty care (TCCC) data.

**Figure 1:**
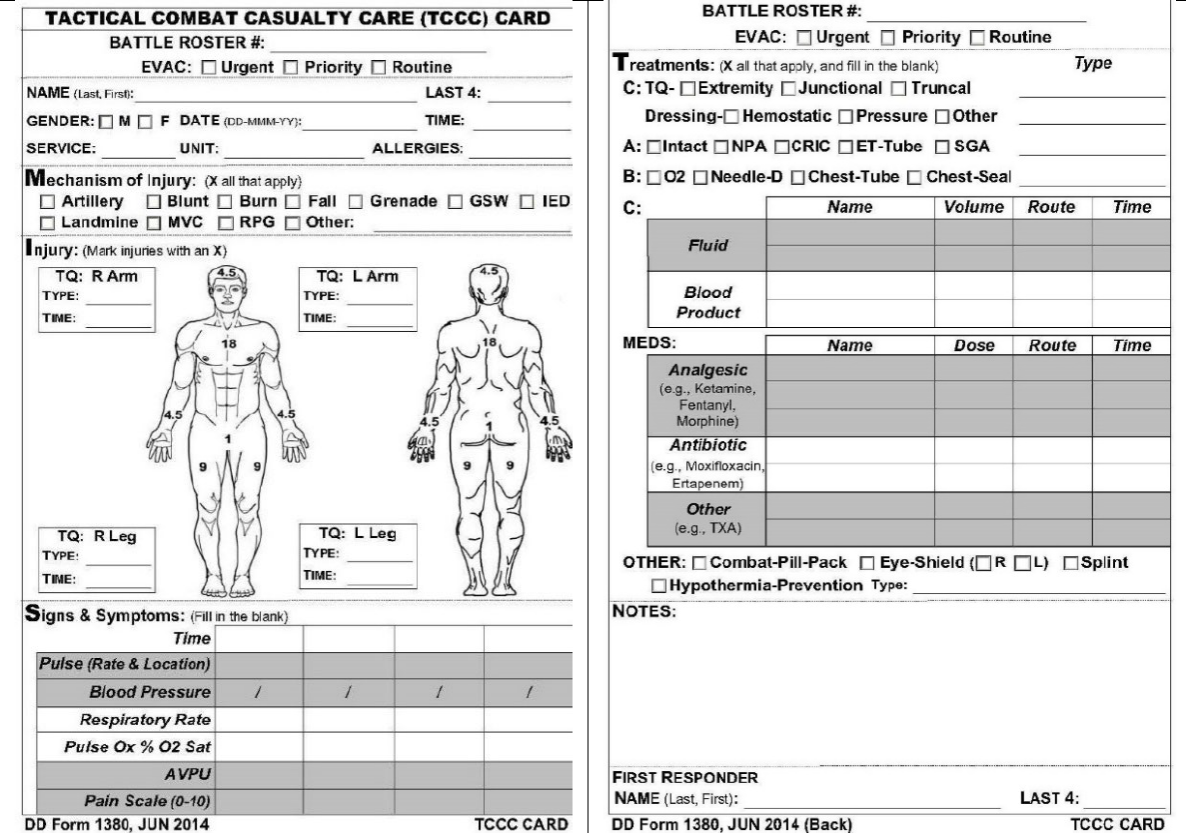
DD Form 1380, the TCCC card.

Historically, TCCC data has been manually collected using the DD Form 1380, also known as the TCCC card (Figure 1). The TCCC card is intended to be filled out by combat medics at point-of-injury (POI) and Role 1 and attached to the casualty’s uniform.

The intent is for this DD Form 1380 to travel with the casualty until they reach their final treatment destination where its contents will be populated into the electronic medical record in the MHS. Current assumptions, which constrain the success of the manual TCCC data capture process, include the following: (1) the DD Form 1380 will be present at the time of the SM’s final treatment destination, (2) the DD Form 1380 will be undamaged and legible to transcribe into the medical record, and (3) there will be abundant resources to transcribe all DD Form 1380 data entries or fields into that record.

Due to the chaotic nature of conflict environments, most of the DD Form 1380s are either incorrectly or inadequately completed. Capturing the medical care provided in these austere venues is secondary to saving lives; however, the need for timely, accurate medical documentation remains for acute care and long-term care management [2-4]. In the near term, this data generates valuable information for higher echelons of care, medical resupply, and logistics systems, and command situational awareness. The long-term benefit is the ability to provide a basis for evaluating quality of care and benchmarking key metrics for quality improvement efforts and to leverage machine learning (ML) and artificial intelligence (AI) to enhance future care delivery in the tactical environment, as well as to inform clinical decision support systems and algorithms deployed in these settings [5].

In May of 2023, the telemedicine and advanced Technology Research Center (TATRC) commenced a new Autonomous Casualty Care (AC2) research portfolio with the initial objective of creating an innovative, trustworthy, reliable solution to enhance TCCC and improve medical documentation in the MHS. TATRC intends to develop a passive (ie, with minimal human effort and distraction), autonomous documentation (AutoDoc) solution of medical care in operational environments comprising a system of sensor suites that passively collect accurate and reliable data about casualty status, care provider actions, and real-time resource usage.

The goal will be for the sensor suites to passively and autonomously document data for a digital DD Form 1380. To accomplish this, TATRC will (1) identify which commercial off-the-shelf (COTS) sensors are most suitable to collect TCCC data elements required to populate the DD Form 1380; (2) conduct human subjects research using participants who perform TCCC skills in controlled, simulated environments; and (3) annotate all sensor suite data collected to build a TCCC dataset for current and future ML and AI algorithms to leverage.

## Methods

### Overview

Since the AC2 mission realignment in May 2023, TATRC has worked in partnership with the United States Army Medical Research and Development Command (USAMRDC) Institutional Review Board Office (IRBO) to ensure optimal human participant protections can be achieved within the scope of our research study. The novelty of developing a study design that leverages sensor suites and simulated data across multiple locations and includes iterative improvements in the technology configuration features due to the agile development process is groundbreaking for the organization. TATRC received its initial human subjects research protocol approval (#M-11057) from the USAMRDC IRBO in February 2024 to conduct this research and will continue to amend the protocol as new collaborators onboard and new methodologies are adapted, as required. Study personnel will follow the protocol requirements including consenting mandates as directed by the IRBO.

Passive data collection using AutoDoc is a research, development, test, and evaluation (RDT and E) project that will be conducted in three separate stages: (1) selection and procurement of sensor suite components, (2) data collection via live human actor simulations, and (3) data annotation.

### Sensor Suites

Individual COTS sensors were bundled into synchronized technology suites designed to capture both patient physiological data and care provider interventions. These passive sensor suites generate data that can be used to generate an electronic DD Form 1380. The categories of sensors used in this research project are listed in Table 1. The COTS sensors selected for the research project’s suite were based on technology maturity level, National Defense Authorization Act compliance, suitability for the operational environment (eg, durability and ruggedization), and ability to extract data from the sensors (eg, nonproprietary data formats). Before leveraging the passive sensor suites in the AC2 research project, the technologies were systematically evaluated through a series of assessments. The sensor suites are worn by consented participants (eg, both the care providers and live simulated patients) in the simulated TCCC scenarios for data collection.

**Table 1:** Categories of commercial off-the-shelf sensors integrated into sensor suites.

Sensor category		Sensor type
**Primary care providers**		
	Audio	Microphone clipped to a collar, lapel, or helmet
	Identification readers	Near field Communication cards attached to items in the care provider’s bag
	Inertial measurement unit	Wrist-worn inertial measurement units to track hand movements
	Mounted video	Traditional video and infrared video
**Simulated Patients**		
	Vital signs monitor	Velcro blood pressure cuff and skin safe adhesive electrocardiogram electrodes leads, adhesive vitals monitoring patch (blood pressure, heart rate, respiration rate, temperature, and SpO_2_^a^), and finger pulse oximeter

^a^SpO_2_: oxygen saturation.

### Data Collection

Live, controlled, simulated scenarios will be used for all passive sensor suite data collection activities. The purpose of conducting live simulation events is to observe participants performing TCCC-related activities. These structured data collection events allow the research team to obtain relevant data for future algorithm development. The purpose of the data collection events is research-specific, and not to educate, assess, or help caregivers self-identify knowledge gaps.

TATRC has identified Department of Defense (DoD) affiliated collaborators to generate the volume of data needed to train the ML and AI. TATRC personnel are currently collecting data at 5 different locations at Fort Detrick, Maryland; Fort Sam Houston, Texas; Fort Indiantown Gap, Pennsylvania; Fort Liberty, North Carolina; and a commercial site in Greenville, North Carolina.

#### Settings

**Figure 2:**
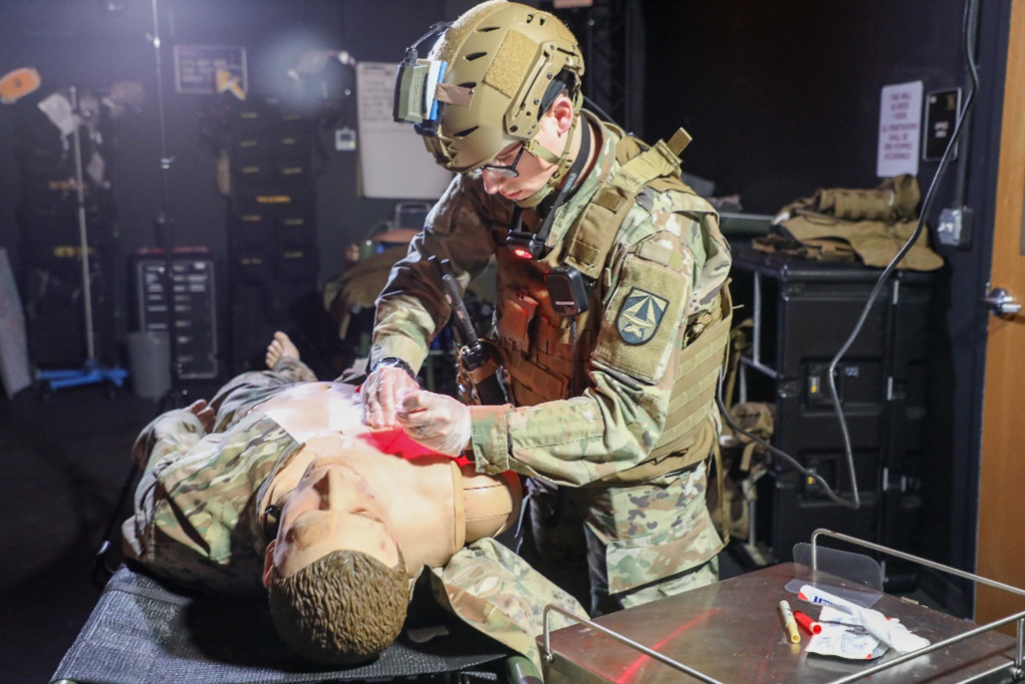
Staged simulation scenario of primary care provider triaging simulated patient (Mannikin).

Live simulation data collection events are conducted at the 5 approved research locations. At each location, a series of physical spaces are dedicated to simulating the prehospital roles of care: POI (casualty collection point or equivalent), Role 1 (battalion aid station or equivalent), Evacuation to Role 2, and Role 2 (Forward Surgical Team or equivalent). In addition, the equipment and medical supplies that are available at each prehospital venue are accessible to the research participants. Data collection locations can create immersive virtual environments, sounds, or other environmental characteristics (smoke, smells, etc) to create a realistic combat environment. In addition, an audiovisual system (AVS) is leveraged to record and archive activities conducted during the data collection events. When data are collected in a formal laboratory venue, the AVS is fixed, but in other locations, a portable AVS is leveraged (Figure 2).

#### Study Participants

Participants will be split into two groups: (1) care provider and embedded participant or (2) simulated patient, based on inclusion criteria. The research team is using video, audio, and kinematic recording equipment to collect first-person data from care providers and embedded participants during the TCCC scenarios. The first-person sensors will record casualty injury patterns, care and tasks provided by the care providers, equipment and supplies used, and other environmental factors. In addition, vital signs monitors, such as finger pulse oximeters, blood pressure cuffs, adhesive electrocardiography patches, and so on, will be placed on the simulated patient participants to record physiological data.

#### Inclusion Criteria

Care provider and embedded participants specific inclusion criteria. This research population group must be a certified provider, as defined below, to participate, ensuring that the simulated data collected reflects data that would be collected by a care provider in a real battle space (Textbox 1). Care provider participants will be split into 2 separate roles during a scenario:

1. Primary care provider (PCP): The PCP is the person providing care to the patient in the scenario and will be required to wear the system of sensors during the simulation.
2. Embedded participant (EP): The EP assists the PCP throughout the scenario by updating vitals, holding items, injecting added pressure onto the PCP, prompting the next steps, and so on.

**Inclusion criteria and exclusion criteria by participant role.**

Inclusion criteria

- Primary care provider or embedded participant
- Are between the ages of 18 and 65 years
- Must have one or more of the following:
  Active tactical combat casualty care certifications for All Service Members, the Tier 1 course
  Working knowledge of combat casualty care skills through medically related training (eg, combat medic or equivalent training)
  Deployment with medical experience in the last 36 months
- There are no preexisting conditions that would negatively impact their ability to provide care for a 20-minute simulated trauma care scenario or work outside for prolonged periods of time (eg, extreme allergies or extreme reactions to sun exposure, etc)
- Simulated Patient
- Age 18 years or older
- Must have the ability to act injured and unconscious for the duration of a 20-minute patient care scenario. The range of injury acting will vary from a state of unconsciousness (ie, lying still) to yelling and writhing in pain

Exclusion criteria

- All participants
- Younger than 18 years
- Currently pregnant
- History of abnormal heart rate (eg, arrhythmias)
- Internal electric medical devices (eg, pacemakers)
- Uncontrolled high blood pressure disorders
- Uncontrolled breathing disorders
- Severe allergies
- Epilepsy
- Primary care provider or embedded participant
- Younger than 18 years or older than 65 years
- Do not hold an active certification for tactical combat casualty care Tier 1
- Simulated Patient
- Younger than 18 years
- Unable to meet the physical requirements of acting as an injured or unconscious patient, such as an inability to act in a predetermined way for up to 20 consecutive minutes or having an adverse response to prolonged time outdoors

#### Exclusion Criteria

For generalized and participant-specific exclusion criteria, please see Textbox 1.

#### Procedures

After recruitment, enrollment, and consent, each participant will be placed into one of 6 simulated scenarios that were scripted for this project. Participants will be paired based on similar levels of expertise described in Bloom’s Taxonomy and then randomized into provider type groups [6].

A total of 6 combat casualty care scenarios were designed and written by TATRC’s combat medic subject matter experts. The injury patterns were developed using common battlefield mechanisms of injury and real-world scenarios. The majority of the TCCC tasks in the scenario correspond to an Individual Critical Task List item for the 68W Combat Medical Specialist, with only a few exceptions. The severity of the casualties differs to capture all triage categories and levels of difficulty. Baseline simulations were developed for each scenario by selecting the appropriate patient mannikin, applying relevant moulage (medical makeup), and by selecting the applicable scene location for POI care. The six scenarios are as follows:

26-year-old male infantryman injured by small arms fire during dismounted patrol. Patient is unresponsive and has a massive hemorrhage from the face or neck and left inguinal area. Enemy threat has been eliminated, and combat lifesaver (CLS) has been attempting to control bleeding for several minutes unsuccessfully.

29-year-old male SM involved in improvised explosive device (IED) explosion. Patient alert to verbal stimuli, and complains of 10/10 pain all over. Obvious right leg below knee amputation. Bleeding from right chest and forehead.

23-year-old male SM injured by small arms fire during key leader engagement. Patient suffered multiple gunshot wounds to the left shoulder, left arm, and left leg. Patient presents with moderate bleeding but alert.

25-year-old male platoon leader injured by mounted IED explosion. Patient has shrapnel and burns to the left arm, leg, and torso with minimal bleeding. Patient is alert and complaining of 10/10 pain. Enemy threat in the area has been eliminated and CLS has been attempting to control bleeding for several minutes unsuccessfully.

35-year-old male platoon sergeant injured by mortar strike. Patient is alert and has right arm amputation below elbow and shrapnel wounds to the right side with moderate bleeding. Patient is alert and complaining of 10/10 pain.

31-year-old male involved in an IED explosion. Patient alert to verbal stimuli with complaints of 10/10 pain on left side of body. Patient has burns to neck and face with stridor. Left side “peppered” with minor shrapnel injuries. Patient is found extricated from vehicle and burning process has been stopped. Scene is absent of enemy activity and no percutaneous transluminal angioplasty treatment has been provided.

Before the simulation commences, the PCP is trained on how to use the sensor suite and if using a simulated patient, the appropriate moulage is applied. The participants are then taken to one of two locations, depending on their assigned scenario: (1) indoor laboratory or training facility: fully controlled environment; and (2) outdoor training facility: partially controlled environment, used for scenes with potential for care under fire.

After arriving at their assigned location, each participant is given a scenario brief. The scenario brief contains information on simulation tasks, conditions, and standards, and allows time for the participants to prep their equipment and supplies or ask any last-minute questions.

**Figure 3:**
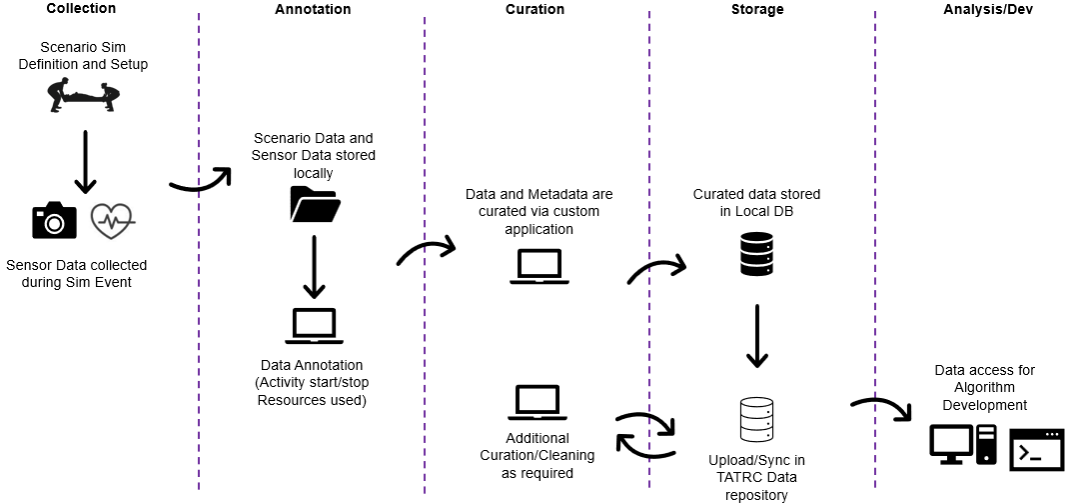
Autonomous documentation flow diagram from simulation data collection to algorithm development. TATRC: Telemedicine & Advanced Technology Research Center.

The data collected from each scripted scenario will differ based on each PCP. Each caregiver will manage the problem set differently based on their personal experiences and knowledge base resulting in individualized outcomes (Figure 3). Only one PCP will manage the simulated patient. The generalized process of how each scripted scenario occurs is as follows:

1. The simulated patient, PCP, and EP are all present at their assigned location.
2. The PCP is trained on the sensor suite technology while the simulated patient is reviewing the script and having moulage applied.
3. The simulated patient, PCP, and EP are given a scenario brief.
4. The start cue is given, and the scenario commences.
5. The simulated patient begins to act out their injuries.
6. The PCP begins performing care to treat the simulated patient, while the EP is standing by. The EP only intervenes if the PCP requests their assistance (eg, to hold equipment or to provide updated patient information), to troubleshoot equipment, or to prompt the PCP.
7. After a determined amount of time, the PCP is notified that evac has arrived.
8. The scenario ends.

From the beginning of the scenario brief to after action reporting, each participant will be engaged in the study for a maximum of 2.5 hours, where 50 minutes is the maximum amount of time a participant can participate in a simulation run.

### Data Annotation

Following the simulation, all simulated TCCC data collected on the sensor suites will be uploaded into TATRC’s secure, PIA-approved data repository. The data are considered “unlabeled” and will require annotation with “labels” for future use in algorithm development.

Unlabeled sensor data must be annotated with labels defining a specific action necessary for algorithm development or “training.” In this case, the object is to build algorithms that, based on sensor data collected in each scenario (input), infer a specific treatment or procedure was performed by providers (output). For example, if a combat medic is placing a tourniquet, then the algorithm must be able to correctly label this specific act using sensor-generated data including start and stop times. Task start and stop times are recorded relative to the simulation start time. The annotation process can be summarized as follows: annotations are applied to each simulation, and 2 annotators review the video and audio resulting from the simulation.

Individually, the annotators record start and stop times for standardized TCCC tasks of interest. Start and stop time identification is guided by pre-established start and stop time markers specific to each task. Examples of the annotations and descriptions of the time points can be visualized in Table 2 below.

1. Comparison and review: task periods (bounded by start and stop times) are compared between the 2 annotators. Any task with less than 80% overlapping between the annotators must be discussed and reannotated by each annotator. If an 80% overlap is still not achieved, another annotator will annotate and review the data. This process will continue until >80% overlap is achieved.
2. Label combination: the final bounds for each task are defined as the outermost stop and start times between the 2 annotators (ie, the union of A and B). The original start and stop times each annotator records, will also be maintained for transparency.

All annotations will be stored in a spreadsheet linked to simulation metadata via a unique simulation ID. The goal of this project is to develop a cloud-based dataset that will enable ongoing and future ML and AI algorithm development for automatic, passive documentation of DD Form 1380 data fields.

**Table 2:** Examples of annotations and descriptions of time points.

Task	Start time (s)	Stop time (s)	Duration (s)
Start of scenario	0	76	76
Tourniquet application	85	142	57
Chest seal application	167	253	86
Nasopharyngeal airway	195	214	19
Treat a casualty for a cold injury	270	346	76
Uses sensor	355	465	110
Documentation	404	579	175
Administer medication	542	579	37
Chest needle decompression	583	612	29
Initiate an intravenous	652	717	65
Uses sensor	681	708	27
Uses sensor	715	738	23
Initiate an intravenous	739	848	109
Administer medication	854	917	63
Administer fluids through an infusion	884	980	96
Documentation	923	954	31
Administer tranexamic acid	956	1048	92
Uses sensor	1010	1050	40
Documentation	1053	1116	63
Bag-valve-mask assisted ventilation	1125	1294	169
Chest needle decompression	1143	1164	21
Documentation	1209	1294	85
End of scenario	1293	1303	10

### Ethical Considerations

#### Ethics Review Approvals or Exemption

This protocol was reviewed and approved by the USAMRDC IRBO, which included human subjects’ ethics review. The approved protocol (#M-11057) was classified a minimal risk to human participants and undergoes annual continuing reviews by IRBO to ensure the safety and ethical considerations of research on all human participants in our research.

#### Informed Consent

Within 48 hours before the simulation scenarios are conducted, one of the research team members will provide the individual with a mailed hard copy or emailed electronic copy of the consent to review and contact them over either a secure telecommunication software or face-to-face, whichever is most convenient for the potential study participant. In either form of meeting, the ombudsperson is also present to explain that participation is voluntary and that the information provided about the research is consistent with the institutional review board (IRB)–approved materials. If over telecommunication software, the research staff member ensures the participant is in a private space and can adequately hear all explanations. If the consent process is in-person, the process takes place in a private area. The trained research effort staff members are completely trained on providing all necessary information to the potential participants and go through the consent form with them. This consent form explains the activities, potential risks, and data collection that the Principal Investigator would like to conduct. The consent form highlights that the data collected in this effort contains footage of the participant’s faces or participants’ voices. As the research team member reviews the consent form with the potential participants, they encourage the participants to ask questions about the data collection specifics.

After the review is complete, the participants are told they may review the consent information up until the day data are collected as the consent form describes and their decision to participate or does not negatively or positively affect their job. The potential participant also is given an opportunity to speak with the ombudsperson alone to discuss any concerns of participation they may have. If the potential participant agrees to participate, she is asked to sign the consent form. Participants will also be asked to sign an optional image release form for use in presentation, promotional, and marketing materials. The participants either sign in person or sign a virtual copy of the consent form and send this back to the research staff member, via methods such as DocuSign or in person. After signing, a copy of the signed consent form is provided to the study participant. The original consent forms are printed and stored in study files in a locked filing cabinet in the TATRC research office. Before leaving the consenting meeting, the volunteers will be reminded that they can reach out to the ombudsperson at any time if they have concerns about participating in this research effort.

#### Privacy and Confidentiality

Care providers and simulated patient participants are assigned a participant ID to be associated with collected demographics, video, and physiological signal recordings. All data are stored in DoD- and HIPAA (Health Insurance Portability and Accountability Act)-compliant safety and security compliant e-storage facilities. All hard copy documents including completed consent forms are stored in locked filing cabinets at TATRC.

The research team collects all video and physiological signal recordings. Following data collection sessions, all recordings are checked within 1-7 days and before media is removed to ensure no nonconsented individual’s identifiable information was not recorded. If any inadvertent recordings were made, the PI or a study team member deletes these segments of recordings. All recordings are stored securely in a DoD-approved, TATRC maintained server or repository including a NAS at Fort Detrick, Maryland. The procedure for consent occurs prior to participants engaging in simulation scenarios. If a person does not consent to data collection, they will not participate in research activities.

One of the main aspects of the data that is collected is that the faces and physiological signatures of the simulated patients are included in the footage of the data. This is needed to be able to accurately train the algorithms and technology that is developing from this effort. This is made abundantly clear to the participants in the consenting process. If they do not consent to their faces being included in this data, they are not allowed to move forward with participating in the collection. Photo Release Waivers for image use in nonresearch activities accompany the consent forms at the start of participation but are entirely optional for participation in the study.

#### Compensation Details

Military volunteers who meet the eligibility requirements and consent to participate in the study have the opportunity to participate in volunteer hours as a participation incentive. After completion of their participation in the study, the participant may request a letter of participation from the research team. The eligibility for volunteer hours toward service recognition will be determined by the units or offices who grant the volunteer hours. The research team will provide the letter of participation in an electronic format to the participant’s email address. This process will be mirrored at all data collection locations. There are no monetary incentives or compensation for this research effort for volunteers.

## Results

**Figure 4:**
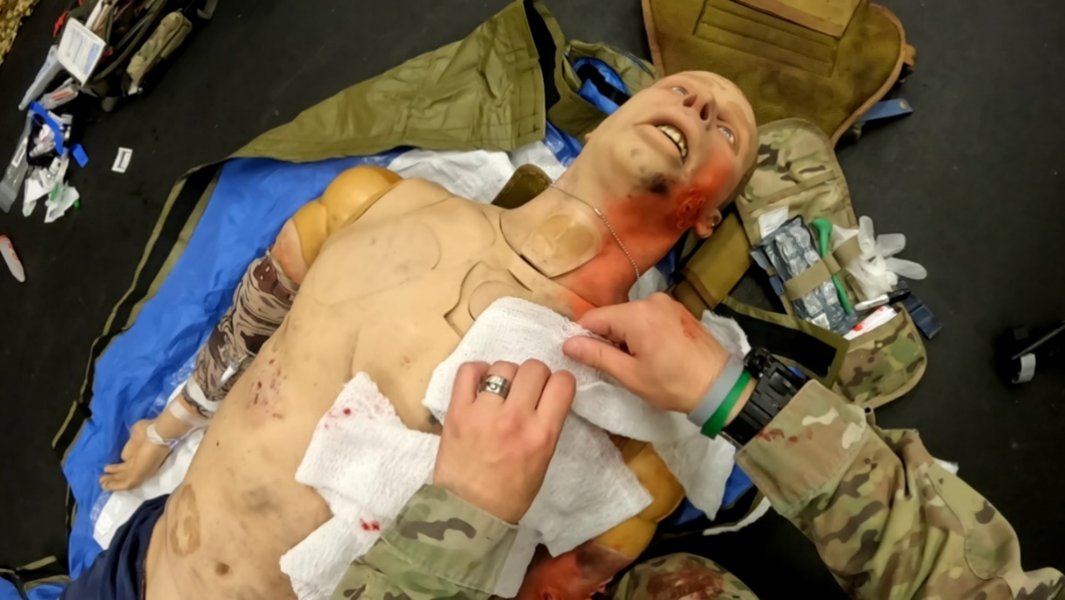
Primary care provider camera view while performing TCCC procedures on simulated patient (Mannikin). TCCC: tactical combat casualty care.

TATRC is on track to collect and annotate approximately 2500 simulation procedures (or tasks) by March 2025. In addition to the data recorded at each TATRC-hosted simulation event, TATRC intends to continue to expand its data repository by acquiring data from similar recorded simulated events at other military and civilian organizations and creating or obtaining augmented or synthetic TCCC data through synthetic modeling environments (ie, digital twin systems). Synthetic modeling environments can generate novel TCCC data to increase the variety and complexity of the datasets. To furthermore diversify the datasets, TATRC plans to collect simulation data from non–TATRC sponsored events (ie, research conferences, operational exercises, etc). Combining both physical and augmented simulation data, TATRC’s aspirational goal is to accumulate approximately 100,000 TCCC simulated procedure or task recordings to populate and generate DD Form 1380 elements by the end of September 2025 (Figure 4). As the annotated simulation procedures are aggregated into TATRC’s data repository, contracted extramural partners will commence algorithm and software development to support TATRC’s goal if automating combat casualty care scene interpretation and patient encounter documentation to significantly reduce manual data entry and enhance the capabilities of combat medics. By September 2025, the extramural algorithm partners intend to complete the machine perception algorithms and associated software to leverage data collected by the AutoDoc suite of sensors to auto-populate all DD Form 1380 elements into a digital XML or JSON format.

## Discussion

TATRC’s AC2 mission focuses on addressing and improving the current reality for combat medics:

A distracted, stressed combat medic managing their way through a chaotic battlefield with multiple casualties surrounds them, with only a bag full of supplies, including one permanent marker and several DD Form 1380 cards.

There are minimal improvements in how the Joint Forces have documented health data beyond our borders, specifically in operational settings in times of conflict, despite the large advancements in data science and technology in the last decade. TATRC’s goal is to replace the current, paper-based process, with modern, passive data collection and automated documentation. The aim is not only to create a more reliable, accurate method for collecting casualty care data but to alleviate the care provider’s cognitive burden in the battle space of this strenuous additional duty.

The methodology for collecting TCCC data may raise questions on why our research team is not leveraging existing or available datasets from historical conflicts. The simple answer is that they do not exist. During the Afghanistan and Iraq conflicts, there were approximately 30,000 documented casualties but only 10% of those casualties had any documentation of prehospital care, and 1% or less had sufficient information to inform care at the next echelon [7]. Similarly, an unpublished 15-month review by the Joint Trauma System (JTS) found that from 172 submitted DD 1380 Forms from the field, more than 50% of them did not meet the criteria for completeness as measured by a quality checklist developed by the JTS. Furthermore, there is a lack of data granularity necessary in medical documentation from the POI based on an analysis of the JTS DOD Trauma Registry database in 2011 by Therien et al [8]. How, when, and where this data was stored remains unknown. These previous publications have illustrated the extreme, nearly impossible, expectation of documenting TCCC while in the battle space. Limiting TCCC data collection to just 1 form and expecting this 1 form to travel with the casualty from the POI to their final treatment destination, without being damaged or lost, is the reason why only less than 1% of DD Form 1380 during the Afghanistan and Iraq conflicts were sufficient to inform the next echelon of care. The result is that there are no existing combat casualty care datasets within the MHS.

TATRC’s solution to this data limitation is using scripted, simulated TCCC scenarios to capture the data. TATRC has partnered with other DoD entities to provide an environment emulating specific battlefield conditions generating realistic TCCC data outcomes. Together, TATRC and partners, are testing and refining the sensor suites while simultaneously diversifying the TCCC dataset and generating the volume of data needed to create trustworthy solutions for care providers across the echelons of care. Although the AutoDoc project primarily focuses on building DD 1380 Forms, the data obtained across all research locations could be used to create an abundance of tools and capabilities, such as algorithms to automate personal health information documentation into an existing electronic health record (eg, MHS). The possibilities to use this data to benefit the warfighter are limitless as long as you can find a way. TATRC is devoted to having this data serve as a foundation for future research, development, test, and evaluation projects across the DoD to improve our understanding of TCCC and allow organizations across the DoD to build upon our work and create innovative tools to automate future healthcare and improve the current standard of care.

## Abbreviations

ACC: autonomous casualty care
AI: artificial intelligence
AutoDoc: autonomous documentation
AVS: audiovisual system
COTS: commercial off-the-shelf
DoD: Department of Defense
EP: embedded participant
HIPAA: Health Insurance Portability and Accountability Act
IED: improvised explosive device
IRB: institutional review board
IRBO: Institutional Review Board Office
JTS: Joint Trauma System
MHS: military health care system
ML: machine learning
PCP: primary care provider
POI: point of injury
SM: service member
TATRC: Telemedicine & Advanced Technology Research Center
TCCC: tactical combat casualty care
USAMRDC: United States Army Medical Research and Development Command

## Data Availability

The data obtained in this study contains PII. According to our IRB protocol, in order to protect the confidentiality of our participants, the Headquarters, Medical Research and Development Command institutional review board (IRB) requires any institution to execute the proper agreements between HQ MRDC IRB and their respective local IRB.
